# Transgluteal vs. Anterior and Posterior Approaches for Porcine Model of Irreparable Acetabular Labral Tear: A Comparative Study on Feasibility and Safety

**DOI:** 10.1155/bmri/7632394

**Published:** 2026-01-24

**Authors:** Wei Dai, Tiao Su, Liu Yang, Xin Chen, Shu Deng, Guangxing Chen

**Affiliations:** ^1^ Center for Joint Surgery, Intelligent Manufacturing and Rehabilitation Engineering Center, The First Affiliated Hospital of Army Medical University, Chongqing, China, tmmu.edu.cn; ^2^ Chongqing Municipal Science and Technology Bureau Key Laboratory of Precision Medicine in Joint Surgery, Chongqing, China; ^3^ Chongqing Municipal Education Commission Key Laboratory of Joint Biology, Chongqing, China

**Keywords:** hip, model construction, porcine, porcine hip conditions, transgluteal

## Abstract

**Objective:**

To compare the surgical feasibility and safety of three approaches (anterior, transgluteal, posterior) for constructing a porcine model of irreparable acetabular labral tear.

**Methods:**

After a cadaveric anatomical exploration in two pigs to define feasible corridors, 36 male Chinese miniature pigs were randomized (1:1:1) to anterior, transgluteal, or posterior approaches for model construction and labral reconstruction. Primary intraoperative outcomes were blood loss, operative time, and a 0–10 surgical exposure score; postoperative complications and survival were assessed over 3 months.

**Results:**

The anterior approach showed greater blood loss and longer operative time than posterior and transgluteal (both *p* < 0.001), while exposure was worst with anterior (*p* < 0.001) and only trended better with transgluteal versus posterior (*p* = 0.056). Postoperative events included sciatic nerve‐related claudication (4/12 posterior), acetabular chondral lesions (3/12 anterior), and poor wound healing (4/12 anterior; 2/12 posterior). One‐month survival was lower in anterior versus transgluteal (*p* = 0.025), with most deaths within 2 weeks; 2‐ and 3‐month survival were also lower for anterior versus transgluteal (*p* = 0.026; *p* = 0.011). At 2 months, survival was lower in anterior versus posterior (*p* = 0.011).

**Conclusion:**

The transgluteal approach appears to be a relatively safe and effective option for constructing porcine hip models; nevertheless, conclusions should be interpreted with caution given the undetermined mortality etiology in the anterior group.

## 1. Introduction

The hip is a ball‐and‐socket joint that functions as the main weight‐bearing joint and part of the spine‐pelvic‐hip complex [1–3]. Hip dysfunction caused by various hip conditions is responsible for pain, stiffness, loss of range of motion, and further reduction of life quality. With the initial disease untreated, the hip will progress to an osteoarthritic condition due to the loss of a normal biomechanical environment. Nevertheless, the pathogenesis and outcomes of related treatment of the diseases like chondral lesion and labral tear remain uncertain because the clinical study is limited. Therefore, animal models are required to reproduce the investigated conditions.

Pigs and humans share high similarities from anatomic, genetic, and physiological perspectives. Due to the various sizes of pigs, we can choose suitable breed and age to perform different kinds of procedures in human medicine such as endoscopy, organ transplantation, catheterization, and neuroimaging [4–6], which are difficult to carry out in other animal models including rodents. Genetically, the size and composition of the porcine genome are similar to those of human beings [7]. Moreover, pigs and humans show remarkable physiological similarity, particularly in the functional characteristics of their organs.

Hence, pigs are favored when the animal model is required to study relevant hip diseases like osteoarthritis and osteonecrosis [8–10]. However, operative procedures are not needed for animal model construction in those studies mentioned above. As far as we are concerned, there is no study reporting a reliable approach for building a porcine model of specific hip conditions including chondral lesions, labral tears, ligamentum teres injuries, and bone defect. In this study, we aimed to propose a safe, efficient, and reproducible approach for the porcine model construction of irreparable acetabular labral tear. An earlier version of this work has previously been published as a preprint on ResearchSquare: Tiao Su, Liu Yang, Guang‐xing Chen. Transgluteal Approach for Porcine Model Construction of Specific Hip Conditions. ResearchSquare, 2022 (doi:10.21203/rs.3.rs-1328959/v1; URL: https://www.researchsquare.com/article/rs-1328959/v1) [11].

## 2. Materials and Methods

The animal protocols for this study were approved by the Laboratory Animal Welfare and Ethics Committee of Third Military Medical University (project number: AMUWEC20211776). Skeletally mature male Chinese miniature pigs, weighing 20–25 kg, were included in the study of porcine model construction for specific hip conditions.

### 2.1. Randomization and Housing Environment

The animals were randomly assigned to experimental groups using a computer‐generated randomization sequence. Randomization was performed to ensure that each animal had an equal probability of being placed in any of the experimental groups. This method minimizes selection bias and balances both known and unknown confounding factors across groups, thus ensuring the reliability and validity of the experimental outcomes. All animals were housed under standardized conditions in a controlled environment with a 12‐h light/dark cycle. The temperature was maintained at 22 ± 2°C with a relative humidity of 50–60%. The animals were provided with ad libitum access to a standard diet and water. The housing conditions were monitored daily to ensure the well‐being of the animals and to maintain a stable environment for the duration of the study.

### 2.2. Anatomical Study

**Figure 1:**
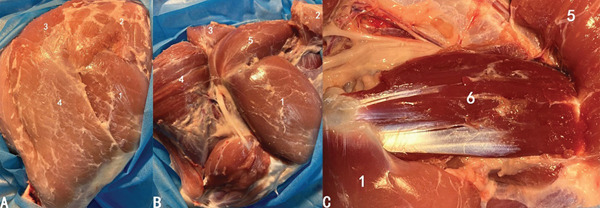
Muscle groups around the hip. (a) The superficial layer. (b) The second layer. (c) The third layer. (1) Quadriceps femoris. (2) Tensor fasciae latae. (3) Gluteus superficialis. (4) Biceps femoris. (5) Gluteus medius. (6) Gluteus profundus.

Two 6‐month‐old pigs were sacrificed to explore the anatomy of the hip including musculature, neurovascular structures, and soft tissues like capsule. We have found that there existed generally three layers of muscle group surrounding hip joints. The superficial layer comprised the tensor fasciae latae, gluteus superficialis, quadriceps femoris, and biceps femoris (Figure 1a). Underneath the tensor fasciae latae and gluteus superficialis lay the gluteus medius (Figure 1b). The distal end of the gluteus medius was the greater trochanter and the dorsal end was the ilium. The gluteus profundus was exposed when flipping over the gluteus medius (Figure 1c).

**Figure 2:**
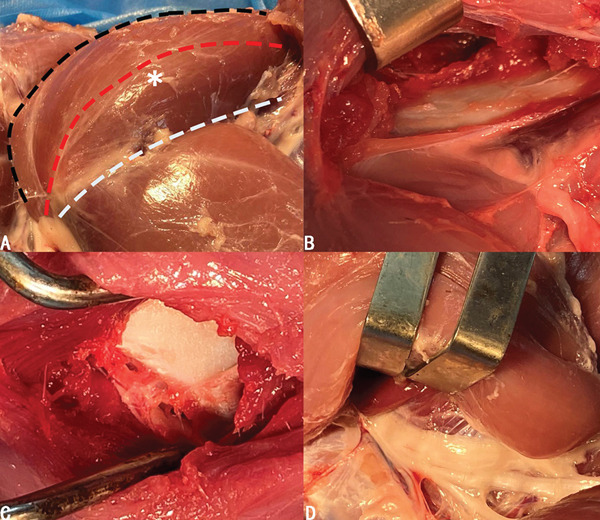
(a) Three possible options to reach the hip joint when facing against the gluteus medius (asterisk): anterior (white dotted curve), transgluteal (red dotted curve), and posterior (black dotted curve). (b–d) Viewing from anterior, intragluteal, and posterior approaches in order.

As mentioned above, the hip joint lay right under the gluteus medius, which was either faced or bypassed when the superficial layer of muscle groups was incised for exposure. We have found that both going anterior and posterior to the gluteus medius could reach the central compartment (Figure 2b,d), in which the gluteus medius was retracted anteriorly or posteriorly with a retractor to obtain exposure. We have also considered bluntly dissecting the muscular part and going right through the middle of the gluteus medius, which provided the best operative exposure and the least surgical tension (Figure 2c). Based on the cadaveric finding, we have come up with three possible approaches to build a porcine model: anterior approach, transgluteal approach, and posterior approach (Figure 2a).

**Figure 3:**
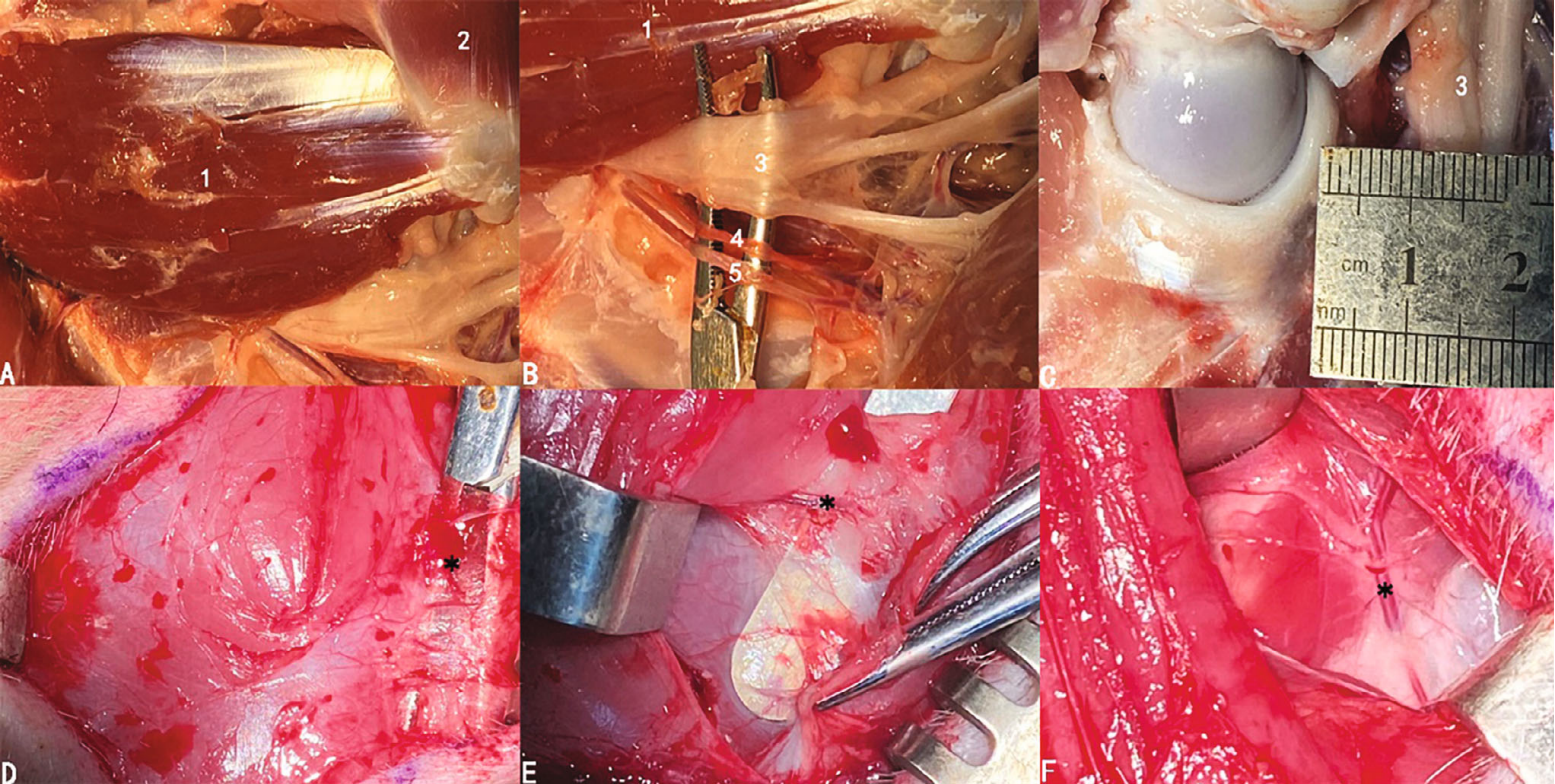
(a) The neurovascular structures of the posterior side of the hip joint. (b,c) The neurovascular structures lie right next to the gluteus profundus and caution must be paid during the procedure due to the close relationship between the hip joint and neurovascular structures. (d–f) Relevant vascular structures (black star) of each layer when using the anterior approach. (1) Gluteus profundus. (2) Quadriceps femoris. (3) Sciatic nerves. (4) Arteria femoralis caudalis. (5) Posterior femoral vein.

The most vital neurovascular structures—the sciatic nerve, arteria femoralis caudalis, and posterior femoral vein—coursed along the dorsal side of the gluteus profundus, all covered by the gluteus medius (Figure 3a,b). The distance between the neurovascular structure and the posterior side of the hip joint was approximately 1 cm (Figure 3c). Therefore, using the posterior approach might increase the chance of injury to major neurovascular structures, especially the sciatic nerve. As for the anterior approach, we identified and ligated intervening vascular branches during the multilayered muscular dissection to reach the central compartment (Figure 3d–f). Thus, extra blood loss would possibly be the underlying risk of the anterior approach. Despite the lower risk of neurovascular injury in transgluteal group based on the cadaveric findings, there might be a potential risk of muscular dysfunction in transgluteal approach.

### 2.3. Animal Model Construction

Thirty‐six male Chinese miniature pigs (6 months old and weighing 20–25 kg) were evenly randomized into three groups according to the approaches they underwent, i.e., the anterior, posterior, and transgluteal groups. All groups were used as models of irreparable acetabular labral tear and received subsequent treatment of labral reconstruction.

#### 2.3.1. Graft Preparation

**Figure 4:**
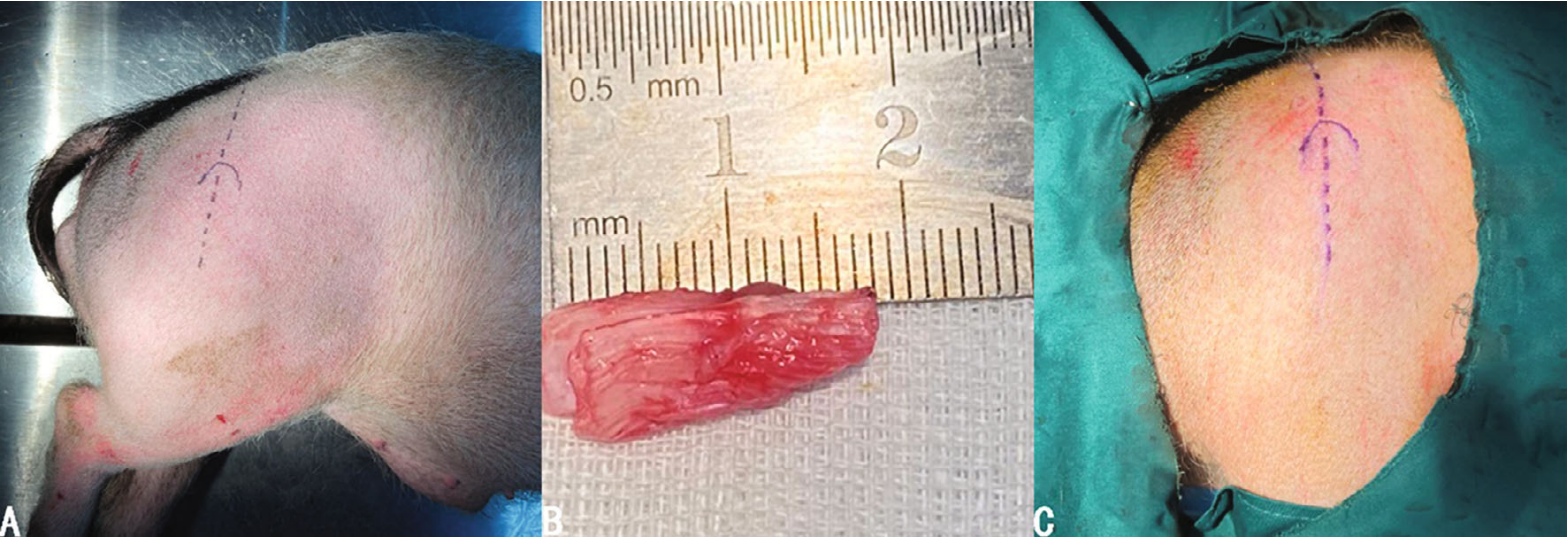
(a) Positioning and surgical incision marking of pigs for operation. (b) Surgical draping. (c) Harvesting of the autologous graft.

The pig was placed in a lateral position on the operating table and surgical incision was marked (Figure 4a). An autogenous Achilles tendon graft was used for this technique. A longitudinal incision was made posterolateral to the ankle in line with the long axis of the tibia. The lateral haft was harvested along the Achilles. The harvested graft was supposed to be approximately 15 mm in length. The graft was carefully cleaned of residual muscular and adipose tissue. Given the inherent tubular configuration of the porcine Achilles tendon, additional tubularization sutures were deemed unnecessary (Figure 4b).

#### 2.3.2. Surgical Techniques

**Figure 5:**
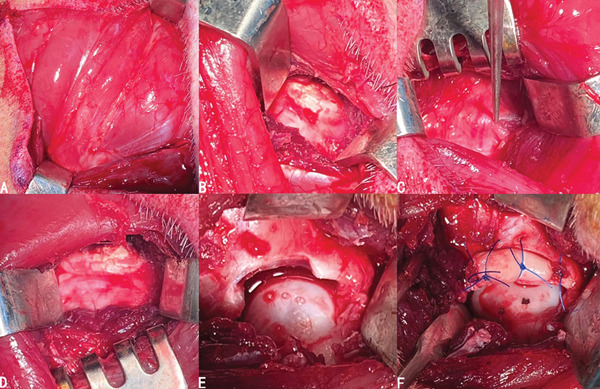
Surgical procedures of model construction. (a) Exposure of the gluteus medius. (b–d) Going anteriorly, posteriorly, and right through the gluteus medius to reach the central compartment. (e) Labral defect after labral resection. (f) The following reconstruction with prepared graft secured to the acetabulum rim.

The pigs were anesthetized by inhaling isoflurane with a concentration of 2.5‐3%. After skin disinfection and draping (Figure 4c), a straight incision was made directly above the greater trochanter. The skin, subcutaneous superficial fascia, and aponeurotic fascia were incised. The tensor fasciae latae was incised between tensor fasciae latae and quadriceps femoris to expose the gluteus medius (Figure 5a). The transgluteal group underwent blunt dissection directly through the musculus gluteus medius to reach the capsule. By contrast, in the anterior and posterior groups, the gluteus medius was not dissected but retracted anteriorly or posteriorly with a retractor to obtain surgical exposure (Figure 5b–d). Following that, the gluteus profundus was exfoliated to incise the capsule and the labrum was exposed (Figure 5e). A 1.0‐cm‐long anterior labrum was resected, and the labral defect was reconstructed with the prepared graft secured to the acetabular rim using nonabsorbable 3‐0 sutures (Ethicon) (Figure 5f).

#### 2.3.3. Postoperative Rehabilitation

Postoperatively, the pigs were injected with penicillin intramuscularly twice to prevent infection. There was no weight‐bearing restriction or immobilization during the recovery period.

### 2.4. Evaluation of Safety and Efficiency

All pigs were followed for 3 months postoperatively, and the surviving pigs were humanely euthanized following institutional guidelines. Intraoperative blood loss and operative time of all pigs were recorded. Surgical exposure was rated from 0 to 10 points, where 10 stands for an excellent operative field, and 0 means exposure is absolutely not enough for operation. At the endpoints of follow‐up, any postoperative complications were recorded and survival curves were analyzed.

### 2.5. Statistical Analysis

All statistical analyses were performed with SPSS (version 21, SPSS Inc., Chicago, Illinois) and GraphPad Prism 7.0 software (GraphPad, La Jolla, California, United States). A *p* value < 0.05 was considered statistically significant. Continuous values were compared between groups with two‐tailed Student *t* tests. Survival curves were analyzed using the Kaplan–Meier method and compared with the log‐rank test.

## 3. Results

### 3.1. Postoperative Complications

Four pigs of the posterior group were found to have claudication due to surgery‐related injury of the sciatic nerve. Chondral lesions in the acetabulum were observed in three pigs of the anterior group, which might be secondary to surgical injury. In addition, poor wound healing occurred in 4 pigs of the anterior group and 2 pigs of the posterior group (Table 1).

**Table 1:** Postoperative complications of all groups.

**Postoperative complication**	**Transgluteal group**	**Anterior group**	**Posterior group**
Claudication	None	None	4
Chondral lesions	None	3	None
Poor wound healing	None	4	2

### 3.2. Comparison of Intraoperative Findings

The blood loss of the anterior group was significantly greater than the posterior and transgluteal groups (*p* < 0.001), while there was no significant difference in blood loss between the posterior and transgluteal groups. The anterior group required significantly longer operative time than the posterior and transgluteal groups (*p* < 0.001), while the operative time of the transgluteal group was similar to that of the posterior group. The surgical exposure points indicated the worst surgical exposure of the anterior group (*p* < 0.001); although the transgluteal group showed a better exposure score than the posterior group, the difference was not statistically significant (Table 2).

**Table 2:** Comparison of intraoperative findings between groups.

	**Trans vs. anterior**		***p* value**	**Trans vs. posterior**		***p* value**	**Anterior vs. posterior**		***p* value**
Blood loss (mL)	87.9 ± 8.9	206.3 ± 3.4	< 0.001	87.9 ± 8.9	88.6 ± 5.4	0.851	206.3 ± 3.4	88.6 ± 5.4	< 0.001
Operative time (min)	32.8 ± 2.5	69.1 ± 2.5	< 0.001	32.8 ± 2.5	33.8 ± 0.9	0.16	69.1 ± 2.5	33.8 ± 0.9	< 0.001
Surgical exposure (point)	7.2 ± 1.1	4.3 ± 0.8	< 0.001	7.2 ± 1.1	6.4 ± 1.1	0.056	4.3 ± 0.8	6.4 ± 1.1	< 0.001

#### 3.2.1. Survival Rates

As shown in Figure 6 and Table 3, the anterior group showed significantly lower 1‐month survival rate than the transgluteal group (*p* = 0.025). Although there was no significant difference, a trend toward lower one‐month survival rate was observed in the anterior group compared with the posterior group. Most deaths occurred within 2 weeks after surgery in all groups (three of six pigs in the anterior group and one of two pigs in the posterior group). Both 2‐month and 3‐month survival rates of the anterior group were lower than the transgluteal group (*p* = 0.026 and *p* = 0.011). There was a significant difference in 2‐month survival rates between the anterior group and posterior group (*p* = 0.011).

Figure 6Comparison of cumulative survival curves in each phase: 1 month (a–c), 2 months (d–f), 3 months (g–i).

**Figure:**
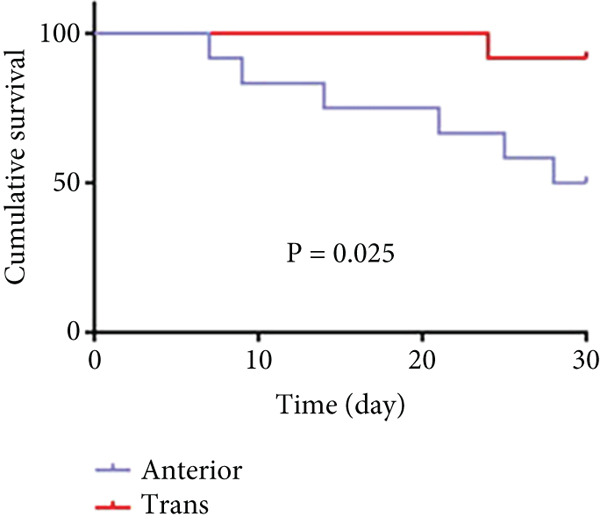
(a)

**Figure:**
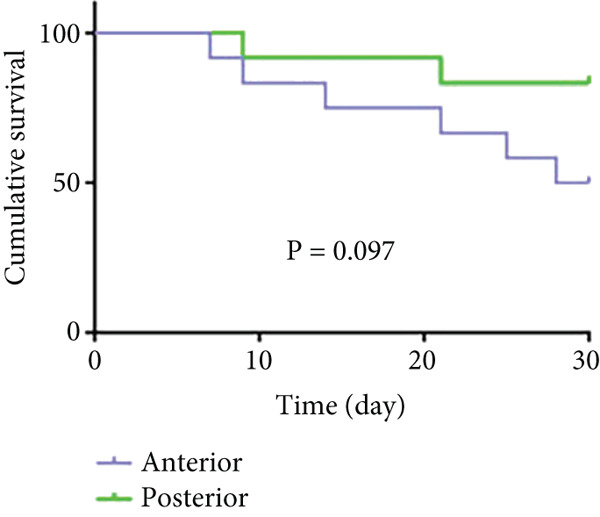
(b)

**Figure:**
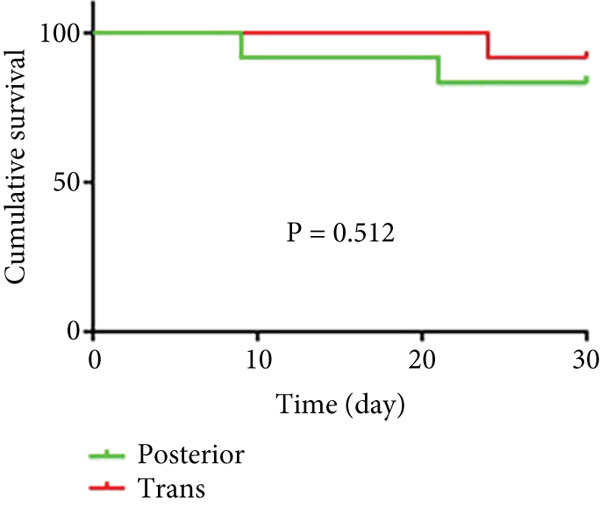
(c)

**Figure:**
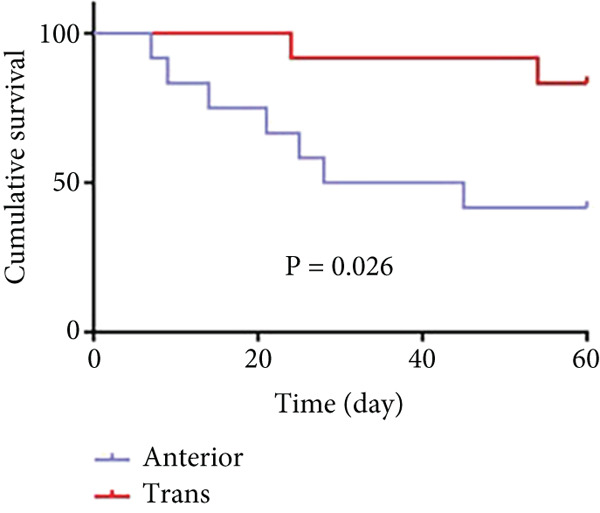
(d)

**Figure:**
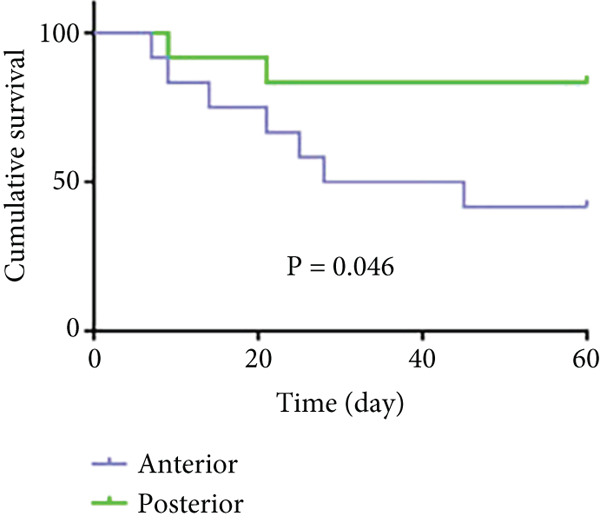
(e)

**Figure:**
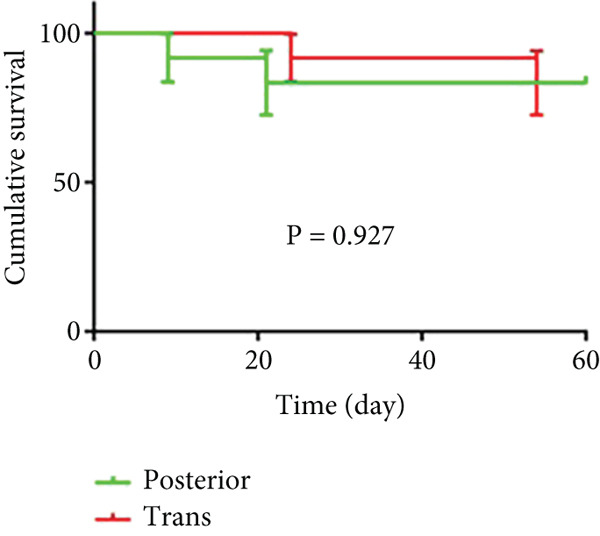
(f)

**Figure:**
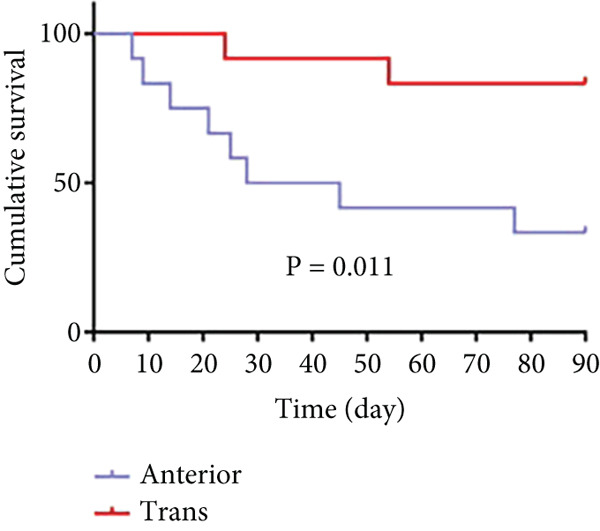
(g)

**Figure:**
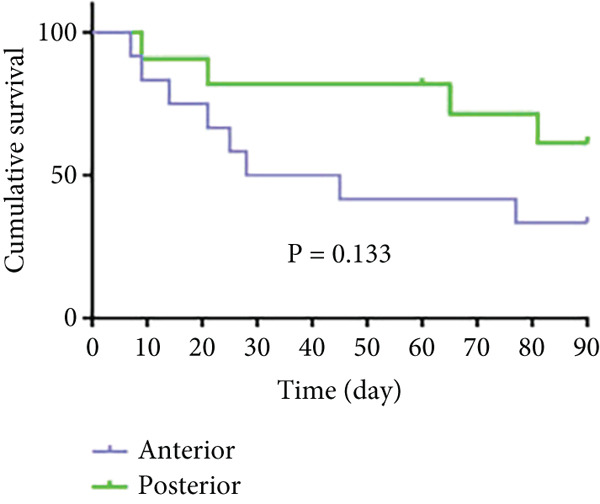
(h)

**Figure:**
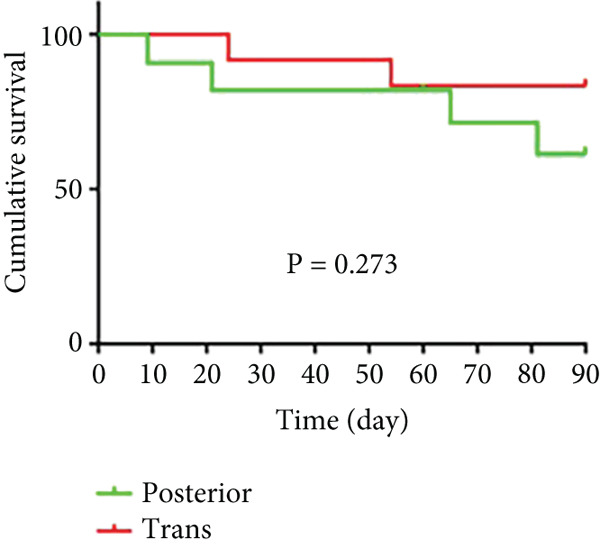
(i)

**Table 3:** Survival rates of each phase in all groups.

**Survival rates**	**Transgluteal group**	**Anterior group**	**Posterior group**
One month	0.9167 ± 0.798	0.5 ± 0.1443	0.8333 ± 0.1076
Two months	0.8333 ± 0.1076	0.4167 ± 0.1423	0.8333 ± 0.1076
Three months	0.8333 ± 0.1076	0.3333 ± 0.1361	0.6136 ± 0.1526

## 4. Discussion

The blood loss and operative time of the transgluteal group tend to be the least, which is mostly attributed to the best surgical exposure for operation. Additionally, there was no abnormal gait observed in the transgluteal group during the follow‐up, indicating a good recovery of gluteal medius. With the anterior approach, excessive surgical tension seemed to obstruct the way to operate in the hip joint, leading to greater operative time and blood loss. Given the close relationship between the posterior side of the hip joint and adjacent neurovascular structures, there is a potential risk of surgery‐related injury of the sciatic nerve, causing claudication. Poor wound healing occurred in both anterior and posterior groups due to the necessity for longer surgical incisions to achieve adequate exposure.

Despite the tremendously increasing understanding of the underlying diseases causing hip pain, the pathogenesis and outcome of relevant treatment remain uncertain. Acetabular labral labrum, for example, is thought to play an important role in the maintenance of suction seal, equal distribution of hip loading, and hip joint stability [12, 13]. Labral tear can alter the biomechanical environment of the hip, leading to premature osteoarthritis and hip pain [14]. With the development of the arthroscopic instrument and surgical experience, the management options have progressed from simple labral resection to labral repair and labral reconstruction over the past decades [15, 16]. However, clinical outcomes of those treatments in human studies remain inconsistent, with several reports indicating a relatively high conversion rate to total hip arthroplasty (THA) [17, 18]. Investigating factors associated with treatment failure remains challenging in clinical studies; therefore, controlled animal experiments are needed to validate the therapeutic efficacy and elucidate underlying mechanisms. Like labral tear, the management of chondral lesions poses great challenge due to the uncertain potential for cartilage repair, and the underlying mechanisms of specific hip conditions such as femoroacetabular impingement, osteonecrosis, and osteoarthritis remain to be fully understood.

As discussed, animal models are essential for investigating the nature of specific hip conditions, including chondral lesions, acetabular labral tears, osteonecrosis, and evaluating treatment outcomes, including osteotomy and osteoplasty for osseous abnormality, and tissue repair or reconstruction for damaged soft tissues. Just as Meurens et al. [18] claimed, animal species were chosen for model building due to their ability of replicating the investigated condition and the similar body reaction with human beings. The extensive homology between pigs and human beings makes the porcine model a possible option for scientific research compared with other species. Furthermore, the porcine model offers sufficient space for surgical intervention, whereas smaller animal hip joints may present technical challenges. However, to our knowledge, there is a lack of published protocols for porcine hip model construction, which could otherwise provide critical insights into poorly understood hip joint mechanisms.

To address this need, we sought to develop a safe and efficient approach for constructing a porcine model of specific hip conditions. Through our study, the transgluteal approach appeared to be the most favorable option among the three possible approaches. Given the anatomic relationship between the gluteus medius and hip joint, going right through the middle of the muscular part provides optimal surgical exposure, which showed a trend toward better exposure compared with the posterior approach, although this difference did not reach statistical significance. Such exposure could enhance procedural efficiency by reducing operative time and minimizing intraoperative blood loss. This might explain the faster recovery and low mortality of the transgluteal group as it renders less trauma and discomfort, providing a better recovery baseline among the three groups. Additionally, the constructed model requires sufficient survival duration to enable development of the targeted conditions [18]. According to our cadaveric findings, the adjacent vascular structures are too close to avoid bleeding from surgical injury bypassing the gluteus medius anteriorly. This likely explains the increased intraoperative blood loss observed in the anterior group compared with the transgluteal group. The extra blood loss of the anterior group might have contributed to postoperative reduced immune function and subsequent respiratory infections, which could partly explain the higher mortality compared with the transgluteal group. However, postmortem examinations were not incorporated into the study design, as the primary objective was to evaluate surgical feasibility and short‐term recovery. Therefore, the exact etiology of mortality remains undetermined, which represents a significant limitation and reduces the certainty of conclusions regarding the specific risks and safety profile of the anterior approach.

Our study showed that the anterior approach was associated with the highest surgical tension among all approaches evaluated. As the gluteus medius starts from the dorsal part of the ilium and courses posterior and backward to end at the greater trochanter, the major muscular part of the gluteus medius obstructs surgical access when attempting an anterior approach to the hip joint. Our data revealed significant temporal inefficiency in the anterior group, with procedures requiring more than 30 min compared with the other two groups. This prolonged operative duration may contribute to the observed lower survival rate and increased incidence of chondral lesions. Although the posterior group demonstrated comparable intraoperative results to the transgluteal group in terms of blood loss and operative time, it was associated with a significantly higher incidence of surgery‐related injury of the sciatic nerve. Nearly half of pigs in the posterior group demonstrated impaired limb elevation and subsequent claudication. Despite the lesser resistance of retracting the gluteus medius, the so close distance (about 1 cm) between the neurovascular structure and posterior side of the hip joint makes it a great challenge to protect the sciatic nerve from injury during operation. Besides, both the anterior and posterior approaches require extending incision for exposure, which is a potential risk factor of delayed wound healing. Notably, no muscular dysfunction of gluteus medius was observed in the transgluteal group, where the integrity of the muscular part was restored. However, this conclusion is based on observational assessment only, and future objective gait analysis will be required to comprehensively characterize functional outcomes and to rule out subtle muscular deficits.

This study still has some limitations. First, the observation period only lasts for 3 months, which precludes assessment of long‐term outcomes, disease progression in chronic models, or the durability of labral reconstruction. Second, we have only chosen Chinese miniature pig as the animal model. Whether the transgluteal approach is suitable for other breeds requires further verification. Planned future investigations include longitudinal follow‐up, multi‐institutional validation with diverse models, and objective gait analysis to fully characterize functional outcomes.

## 5. Conclusion

In this study, the transgluteal approach showed several advantages: low blood loss, less operative time, faster recovery, and high survival rate. Therefore, it could be a safe and efficient option for porcine model construction of specific conditions.

## Ethics Statement

The animal protocols for this study were approved by the Laboratory Animal Welfare and Ethics Committee of Third Military Medical University (project number: AMUWEC20211776).

## Conflicts of Interest

The authors declare no conflicts of interest.

## Author Contributions

Wei Dai and Tiao Su share the co‐first authorship.

## Funding

This study is supported by the Joint Project of Chongqing Science and Technology Bureau and Chongqing Health Commission, 2020MSXM054; the National Natural Science Foundation of China, 10.13039/501100001809, 82372365; and the Chongqing Natural Science Foundation Innovation Development Joint Fund Project, CSTB2022NSCQ‐LZX0066.

## Data Availability

The data that support the findings of this study are available upon request from the corresponding author Guangxing Chen, by email cgx7676@tmmu.edu.cn.
